# Development of a diabetes self-management + mHealth program: tailoring the intervention for a pilot study in a low-income setting in Mexico

**DOI:** 10.1186/s40814-020-0558-7

**Published:** 2020-02-14

**Authors:** Robin Whittemore, Mireya Vilar-Compte, Soraya Burrola-Méndez, Annel Lozano-Marrufo, Roberta Delvy, Mariana Pardo-Carrillo, Selene De La Cerda, Ninfa Pena-Purcell, Rafael Pérez-Escamilla

**Affiliations:** 1 Yale School of Nursing, 400 West Campus Drive, West Haven, CT 06516 USA; 2 Universidad Iberoamericana, Prolongación Paseo de Reforma 880, Lomas de Santa Fé, 01219 Mexico City, Mexico; 3 Texas A & M University, 2251 TAMU Mailstop, College Station, Texas 77845 USA; 4 Yale School of Public Health, 60 College Street, New Haven, CT 06510 USA

**Keywords:** Type 2 diabetes self-management, Theory-based text message, HAPA framework, Mexico

## Abstract

**Background:**

Type 2 diabetes (T2D) is a public health pandemic disproportionately affecting low- and middle-income countries. The purpose of this formative research was to adapt evidence-based diabetes self-management education programs to the context of Seguro Popular clinics in Mexico. A theory-based mHealth (pictorial text messaging) component was developed.

**Method:**

Our formative research and development of the program protocol consisted of six phases: (1) interviews and focus groups with stakeholders on the challenges to T2D management, curriculum content needs, and the use of mHealth as a supplement to a DSME program; (2) review of the theoretical underpinning, curriculum, and interactive strategies of four evidence-based DSME programs and modification to meet the needs of adults with T2D and systems of care in Mexico City; (3) development of theory-based illustrated text messages; (4) evaluation of text messaging acceptability and access in adults with T2D via focus groups; (5) development of program manual; and (6) development of a training program for health care providers.

**Results:**

The ¡Sí, Yo Puedo Vivir Sano Con Diabetes! included 7 group-based weekly lessons; simple, interactive content; weekly empowerment messages; video novellas; group activities; and goal setting. Adaptations to the cultural context of Mexico included content/activities on diabetes etiology (addressing cultural misconceptions), nutrition (indigenous foods and plate method), self-blood glucose monitoring, and diabetes-related stress/coping. We used the Health Action Process Approach to guide the text message development, which posits that adoption, initiation, and maintenance of health behaviors require the development of intentions, plans, coping, and self-efficacy. Our final text message bank consisted of 181 messages. There were approximately 20–30 messages for each process of behavior change (e.g., action planning, maintenance self-efficacy) and 30 messages for each content topic (e.g., eating healthy, physical activity). There were 96 messages that were illustrated. Training materials were also developed.

**Discussion:**

We used a systematic approach, collaboration with stakeholders, and a well-established behavior change theory to develop an evidence-based intervention to an international context and system of care. Collectively, this process has the potential to enhance the feasibility, acceptability, and efficacy of the program.

## Background

Type 2 diabetes (T2D) is a global health concern with persons in low- and middle-income countries affected disproportionately [1]. Based on 2016 data, the prevalence of T2D in Mexico was estimated at 15.9%, one of the highest in the world [2]. T2D is the second leading cause of death in the country, with direct costs of more than 7 billion dollars in 2011 [3, 4]. Of considerable concern is that, despite having access to medical care, 70% of adults with T2D living in Mexico City have poor glycemic control [glycated hemoglobin (A1C) > 7.0%] with 56% demonstrating extremely poor control (A1C > 11%) [5]. T2D self-management has also been shown to be suboptimal [4]. The overarching goal of our project was to develop and evaluate a novel diabetes self-management education (DSME) program for adults with limited resources in Mexico and improve behavioral, psychosocial, and metabolic outcomes.

Adults with T2D in Mexico may not have access to high-quality DSME programs and often lack knowledge and understanding about T2D etiology and self-management. Many Mexican adults with T2D believe that the primary etiology of T2D is extreme fright or stress [6–8]. This health belief is a potential barrier to T2D self-management, as many Mexican adults with T2D do not associate diet and physical activity with the illness [4]. Low health literacy, low socioeconomic status, use of herbal remedies, psychosocial comorbidities, and limited communication with providers have also been reported among the Mexican population living with T2D [9, 10]. Thus, it is not surprising that self-management is sub-optimal in the majority of adults with T2D in Mexico and that DSME programs are warranted among this vulnerable population.

Mexico’s social security system provides health care for formally registered wageworkers and their dependents. In 2003, a public health insurance system, *Seguro Popular*, was instituted to provide care to individuals not covered by the social security system. Adults who receive care in the Seguro Popular system have a mean age of 44.7 years, are 57% male, have low income (66.9% are in the 3 lowest socioeconomic quintiles), and 12.4% report a diagnosis of T2D [11]. In the Seguro Popular clinics, physicians, nurses, and community health workers provide T2D care which consists of medical visits, referrals to specialists and nutritionists when needed, laboratory testing, and free medicines [12]; however, the provision of diabetes self-management education (DSME) is insufficient [13]. In 2001, the Mexico Health Department implemented a health education program in Mexico for adults with T2D, the “Mutual Help Groups” (GAM—acronym in Spanish). However, data from 2012 indicate that there were 7059 GAM groups with 172,595 beneficiaries, but only 30% of these groups were certified by the Mexico Health Department [14]. Therefore, the overarching goal of our project was to address this gap in services.

There is a widespread use of mobile devices in Mexico. More than 80% of adults in Mexico own a cell phone and text messaging use in Mexico is high at 82–89% [15, 16]. In 2018, the National Institute of Statistic and Geography reported that there were 69.6 million smartphone users in Mexico, from which 93.4% accessed the Internet through their device and 58% of them downloaded and used text messaging apps [17]. Adults aged 25–44 years of age account for 53.6% of cellphone users, while adults 45 years or older account for 29.4% of users. With respect to socioeconomic status, 58.9% of adults from the low socioeconomic strata use cellphones, equivalent to almost 9 million people [18, 19]. Thus, we supplemented the DSME program with behavior change theory-based text messaging (mHealth) as a means of communicating strategies to support self-efficacy and health behavior change, further enhancing self-management of T2D.

In this paper, we describe the systematic process we undertook to develop a DSME + mHealth program for adults with T2D receiving health care in the Seguro Popular clinics in Mexico City—*¡Sí, Yo Puedo Vivir Sano Con Diabetes!* to inform a future pilot study.

## Methods and results

Our formative research and development of the program protocol consisted of six phases: (1) interviews and focus groups with stakeholders on the challenges to T2D management, curriculum content needs, and the use of mHealth as a supplement to a DSME program; (2) review of the theoretical underpinning, curriculum, and interactive strategies of four evidence-based DSME programs [20–24] and modification to meet the needs of adults with T2D and systems of care in Mexico City; (3) development of theory-based illustrated text messages; (4) evaluation of text messaging acceptability and access in adults with T2D via focus groups; (5) development of program manual; and (6) development of a training program for health care providers. Each phase of the process will be discussed in more detail.

### Phase 1: Interviews and focus groups with stakeholders on challenges to T2D management, curriculum content needs, and the use of mHealth

Institutional Review Board approval was obtained from Yale University and *Centro de Análisis y Medición del Bienestar Social* Ethics Committee in Mexico City. All participants of focus groups or interviews provided written informed consent.

We used an interpretive and participatory method with a collaborative team of diabetes experts in the USA and Mexico, clinic administrators, physicians, nurses, and adults with T2D in Mexico City to determine challenges to T2D management, curriculum content needs, and the use of mHealth as part of a DSME program in Seguro Popular clinics. We also explored how the cultural context, family, and health system shape beliefs and practices, and deliver or support care related to T2D. This qualitative study has been published [25]; in the following section, we summarize the main points that informed our intervention development.

Common challenges to T2D self-management were identified by adults with T2D and providers. Personal challenges included (1) cultural beliefs on T2D causation and treatment; (2) challenges to lifestyle modification; (3) lack of access to healthy food, medications, and/or supplies for diabetes management; (4) lack of family support or competing demands; and (5) mental health issues. These personal challenges to T2D self-management and perceived educational needs for adults with T2D are identified in Table 1. Both adults with T2D and providers discussed the need for more information on the cause of T2D, the treatment (medication, diet, and exercise), the cause and prevention of complications, and the need for psychological support. Providers also identified the importance of addressing cultural beliefs/misconceptions, the use of alternative medications, the importance of medical appointment attendance and ongoing laboratory tests, how to use glucometers, and targeted blood glucose levels.

**Table 1:** Challenges and educational needs of adults with T2D

Challenges	Educational needs to be addressed in the intervention
Cultural beliefs on T2D causation and treatment	Cause of T2D Treatment of T2D Cause and prevention of complications
Challenges to lifestyle modification due to low health literacy, difficulty changing established habits, perceived lack of symptoms/personal agency	Healthy eating and physical activity recommendations Simplified content, reinforce content over time Have atmosphere so participants comfortable asking questions Use of images Explore personal strategies to fit lifestyle changes into the life Build confidence in making lifestyle changes
Lack of resources	Uncomplicated and inexpensive meal plans Emphasize the importance of medical appointments/lab work Provide glucometers/strips and education on how to use/how to interpret
Lack of family support	Invite family to sessions Emphasize the importance of healthy eating and exercise for all members of family
Mental health issues	Psychological support/referral Stress management and coping with T2D

Providers also gave recommendations on how the content of the intervention should be presented, such as the use of simple messages, repetition to reinforce knowledge acquisition, and an atmosphere where participants felt comfortable asking questions (Table 1). They also recommended providing adults with T2D-specific strategies on how to fit lifestyle change into their lives, the importance of including family attendance at sessions, and the need to build confidence in the ability of adults with T2D to improve their health through diabetes self-management.

Although the use of mHealth and text messaging was positively received by adults with T2D and providers, concerns were raised regarding access to cell phones, the ability to access text or image messages, comprehension of messages, and ongoing interest in messages over time (Table 2). Recommendations included assessing the ability of adults with T2D to use text messaging on their personal cell phones, the importance of simplified colloquial language in text messages, and the need to avoid language that could be interpreted as overly prescriptive (e.g., “you need to…you should”).

**Table 2:** Text messaging challenges to consider in the intervention

Challenge	Recommendation
Access to cell phones—may not have a cellphone or may not be able to receive text and picture messages	Provide booklet with all daily text messages
Ability to use text messages—may not know how to use text messaging or download image	Assess the technology focus group Provide education on how to use text messages on their cell phone upon enrollment to study
Comprehension of messages—may have low reading ability	Text messages at 3rd–4th grade reading level Use images for the majority of text messages Use colloquial language
Interest in messages over time—may lose interest, stop checking messages, or get annoyed at messages telling them what to do	Have illustrations be empowering and uplifting Use encouraging vs. prescriptive language

### Phase 2: Review the theoretical underpinning, curriculum, and interactive strategies of evidence-based DSME programs and modification to meet the needs of adults with T2D and systems of care in Mexico City

We subsequently reviewed the theoretical underpinnings, content, and interactive strategies from four evidence-based DSME programs developed and evaluated for Latino adults with T2D to develop our program: *¡Sí, Yo Puedo Vivir Sano Con Diabetes!.* Specifics about each program are highlighted in Table 3 [20–23].

**Table 3:** Development of curriculum and activities

Evidence-based DSME program	Topics incorporated into *¡Sí, Yo Puedo Vivir Sano Con Diabetes!*
*¡Si, Yo Puedo Controlar mi Diabetes!* [20]	Diabetes self-care steps Glucose self-monitoring Medication self-management Food label reading Motivation Goal development
T2D DSME program for the older adults in Mexico City [23]	Contextual aspects about T2D in Mexico Diabetes knowledge Nutrition counseling Glucose self-monitoring Adherence to medical treatment Strengthening the relationship with their health provider Emotion recognition and management
Diabetes Among Latinos Best Practices Trial (DIALBEST) [21]	Diabetes knowledge Glucose self-monitoring Food label reading Physical activity Social support
Community Health Worker Assisting Latinos Manage Stress and Diabetes (CALMS-D) program [22]	Stress management strategies Eliciting social support Guided relaxation exercises

¡Sí, Yo Puedo.*¡Sí, Yo Puedo Controlar mi Diabetes!* is a DSME program that was developed to target the unique needs of Spanish-speaking Hispanic/Latinos with low health literacy in the Texas border communities in the USA. It is based on social cognitive theory [26] and consists of six group-based weekly lessons (plus an initial orientation session) with content on understanding T2D as well as blood glucose monitoring, nutrition, exercise, and medication self-management. The primary aim of the program is to empower participants to be active in their care. Experiential learning, modeling, video novellas (stories about adults with T2D), empowerment phrases, goal setting, and group activities are used to improve self-efficacy and promote self-management of T2D. The program has been effective in reducing A1C levels, improving health behaviors, and increasing self-efficacy in Hispanic adults with T2D [20].

#### T2D DSME program

The T2D DSME program for the older adults in Mexico City was developed to target the unique needs of older adults in the community setting. It was based on the theory of planned behavior and in prior models of literacy among older people [27]. Social workers with training in aspects of psychology and geriatric population, nutrition, and education in diabetes provided 20 group sessions of 1.5 h at two community groups for older adults with T2D (> 60 years of age) in Mexico City. The 20 sessions lasted approximately 6 months. The program focused on six main areas: nutritional counseling, glucose self-monitoring, adherence to medical treatment, emotional management, and diabetes education and empowerment targeting the needs of older adults. Through a pre-post analysis, the intervention was considered to be effective in improving glycemic control, self-efficacy in T2D self-management, and self-efficacy for healthcare provider interaction [23, 24].

#### The Diabetes Among Latinos Best Practices Trial program (DIALBEST)

The DIALBEST program was based on stages of change and motivational interviewing theories to provide problem-solving support to Latino adults with T2D and promote positive health behaviors of T2D self-management [21]. Trained community health workers provided approximately 17 home-based sessions to enhance motivation for behavior change, elicit social support, and gain skills in T2D management. Sessions included nutrition counseling including food label reading in supermarkets and healthy cooking), glucose self-monitoring, medication adherence, and physical activity taking into account the social determinants of health. The DIALBEST program was effective in improving glycemic control at 12 and 18-month follow-up.

#### The Community Health Worker Assisting Latinos Manage Stress and Diabetes (CALMS-D) program

CALMS-D was based on theories of stress and coping, adaptation, and mindfulness to improve stress management in Latino adults with T2D. Trained community health workers provided eight group-based sessions on stress, coping, managing emotions, and eliciting social support. Relaxation skill training was provided in the sessions, and a compact disc of guided relaxation exercises was given to all participants for daily usage. Significant improvements in symptoms of depression, anxiety, and self-reported health status were seen in CALMS-D participants compared to a diabetes education group. In addition, increasing attendance at CALMS-D sessions was associated with greater improvements in glycemic control and diabetes distress [22].

In order to develop a DSME program for delivery in the Seguro Popular clinics in Mexico, we incorporated relevant theoretical underpinnings, educational content, and interactive strategies based on the aforementioned programs to meet the needs of adults with T2D with limited resources, expertise of providers, and systems of care. Social cognitive and empowerment theory informed the group-based interactive processes in sessions and the health action process model informed the development of the daily text/picture messages (Table 4).

**Table 4:** Theoretical Underpinning of *¡Sí, Yo Puedo Vivir Sano Con Diabetes!*

Social cognitive theory (Bandura, 1986) [26]	Empowerment theory (Zimmerman, 1995) [28]	Health Action Process Approach Model (HAPA) (Schwarzer, 2008) [29]
Interactive group sessions Role modeling Incremental goals Build on small success Encouragement by program leaders Stress management activities	Activities and discussion to enhance perceived control and motivation Empowerment phrase of the week	Build an intention to change Action planning Coping planning Activate social support Build task, coping, and recovery self-efficacy through daily text messages

Once the outline for each session was approved by the research team, the content and educational materials were developed. All content and activities were provided in Spanish. Health messages were simplified and numerous visual aids were used (e.g., pictorial handouts and homework sheets) to address low health literacy. Each session included an empowerment phrase of the week, goal setting for the next week, and a stress management activity (Table 5).

**Table 5:** Educational content of *¡Sí, Yo Puedo Vivir Sano Con Diabetes!*

Session	Topics and activities
Orientation	Introduction to the program, introduction of group members, expectations of participants, and review the content of each session
Session 1	Understanding diabetes and need for blood glucose monitoring, healthy eating, physical activity, and taking medication Group discussion on common cultural misconceptions about diabetes (e.g. diabetes is caused by a scare or “Susto” in Spanish), eating fruit is wrong, only older adults can have diabetes, insulin treatment is the prelude to death Understanding healthy eating using “The smart plate” for people living with diabetes Stress management activity: Identifying emotions and physical reactions caused by stressful situations, breathing relaxation exercise Goal setting for diabetes self-management
Session 2	Self-monitoring blood glucose: Demonstration and practice of glucometer use, interpreting glucose levels with a problem-solving approach, identifying the effect of different foods on glucose levels Stress management activity: Identifying emotions, mindfulness exercise and breathing relaxation technique Goal setting for diabetes self-management
Session 3	Explanation of food groups, carbohydrates, and importance of a balanced meal. Relationship between carbohydrate intake and blood glucose levels Food measurement: Learning how to measure portions for the different food groups and distributing portions throughout the day Menu planning with limited resources: Selecting seasonal produce, highlighting healthy traditional Mexican dishes using readily available products Stress management activity: Identifying emotions to develop individualized stress coping strategies, progressive muscle relaxation exercise Goal setting for diabetes self-management
Session 4	Physical activity: Benefits, precautions, understanding the safe range of glucose levels to perform physical activity, types of physical activity (aerobic, strength), and recommended levels of physical activity for adults living with diabetes Classroom physical activity game: Ball game with musical cues and diabetes-related trivia Identifying symptoms for hypo and hyperglycemia during physical activity and hypoglycemia emergency management Stress management activity: Mindful breathing exercise Goal setting for diabetes self-management
Session 5	Communication with health care professionals: group discussion guided by a novella on strategies to improve communication with health care professionals and family members Common misconceptions about diabetes medicines: Guided group discussion and video of these issues Reading food labels and choosing the better option Stress management activity: Guided visualization meditation exercise Goal setting for diabetes self-management
Session 6	Review of diabetes self-management steps and stress management strategies Mindful eating exercise Preventing diabetes complications and taking control of diabetes: Foot care, dental prophylaxis, and diabetic retinopathy overview

### Phase 3: Development of theory-based illustrated text messages

The *¡Sí, Yo Puedo Vivir Sano Con Diabetes!* program was supplemented by theory-based illustrated text messaging provided daily for 6 months to promote understanding of T2D self-management, enhance self-efficacy, and provide support for self-management goals. Inspired by the success of a text message program to improve breastfeeding in an underserved population [30, 31], our research team developed a theory-based text and picture bank aligned with our DSME program. The Health Action Process Approach (HAPA) framework was used to tailor text messages to processes of behavior change—risk awareness, behavior change plans, behavior initiation, behavior maintenance, and prevention of relapse. Providing support for these processes is hypothesized to enhance self-efficacy, self-management, and subsequently health outcomes [21]. In the HAPA model, development of perceived self-efficacy at each stage of health behavior change is critical to initiating and maintaining new health behaviors [32, 33].

Messages were written at the 3rd–4th grade reading level with many messages including simple pictures to enhance understanding. Adding pictures to health messages increases retention, comprehension, and adherence, particularly in adults with low health literacy [34]. To develop the illustrations for the text messages, a graphic artist was provided information on the goal of the project and our overall vision for the text messages to be positive and empowering in order to promote feelings of personal agency and confidence in diabetes self-management. For each text message, the artist provided two-three pencil sketches of text messages, which were reviewed by our research team for cultural relevance and perceived participant understanding following an iterative consensus approach. Detailed notes were provided to the artist for recommended changes (e.g., type of food, number of people in the image). The artist then created an illustration of the text message in color that was reviewed and finalized by the research team. See Table 6 for an example of a text/image messages aligned with each process of behavior change.

**Table 6:**
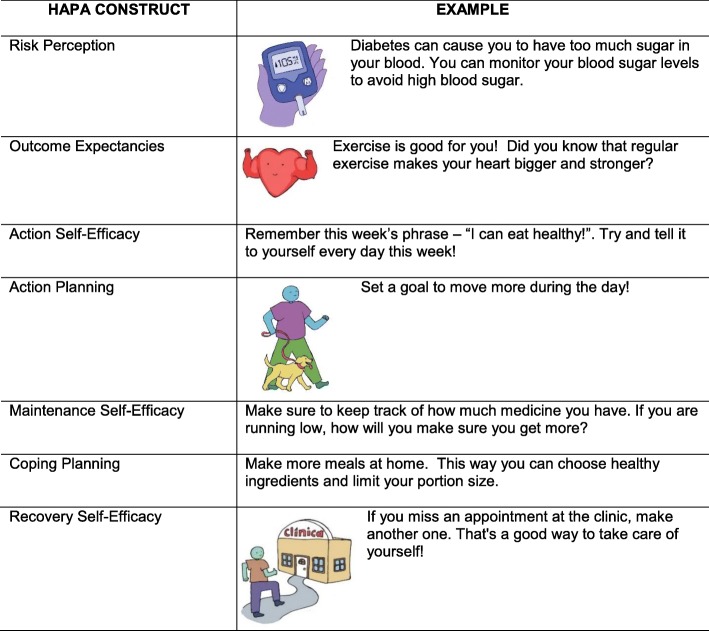
Examples of text messages aligned with HAPA model

For our pilot study, we will align text messages with participation in the program—providing messages on raising risk/benefit awareness at the beginning of the program, moving to messages to promote behavior change planning and behavior initiation (2nd group class), and behavior maintenance/prevention of relapse (final group class). Starting in the sixth week and continuing for up to 6 months, text messages will be developed so that participants receive messages targeting all phases of behavior change, with an emphasis on maintenance/prevention of relapse messages. In future research, we will build algorithms to assess where an individual is in the process of behavior change and tailor messages accordingly.

### Phase 4: Evaluation of text messaging acceptability and access in adults with T2D

After developing approximately 25 text/illustrated text messages, we conducted focus groups with adults with T2D at two Seguro Popular Clinics in Mexico City to elicit their feedback on their understanding of text message content, their perceptions of the images, and their ability to receive and download text and image messages. Participants could participate in one or both of these focus groups. Written informed consent was obtained from participants, and they were compensated with 100 pesos as a thank you for their time for each focus group that they participated in.

For the first focus group, each participant was provided a workbook with a single text message on a page (some text messages, some image messages). The moderator went through the booklet and asked participants to provide their opinions on the presentation of content (words/images) and their comprehension of the messages. The moderator showed the image or text message to the group and asked, “What do you see/read?” “What does this mean to you?” “Does this apply to you?” “How would this message help you to think about your diabetes?” A second research assistant recorded participant responses for each message, which were summarized into a table, with comments provided for each text/image message.

There were nine adults with T2D who participated in this stage of the text messaging evaluation with the following characteristics: 77.78% female, median age 50 years [interquartile range (IQR) 13], mean age 52.33 years [standard deviation (SD) = 10.47], 88.89% had less than high school education, 88.88% married or in cohabitation, mean A1C 9.13% (SD = 1.07), and had a median of three comorbidities. Overall, participants understood the intended meaning of the majority of the text messages and felt that the messages were positive and helpful. There were several text messages that they perceived did not apply to them or conveyed a misconception that needed to be addressed in the curriculum (see Table 7 for examples). We intentionally created images of people of varying body sizes; however, several participants felt that the people in the images “seemed fat” which was not motivating for them. The results of this focus group were used to guide revisions of these messages as well as the development of the rest of the text messages and images.

**Table 7:**
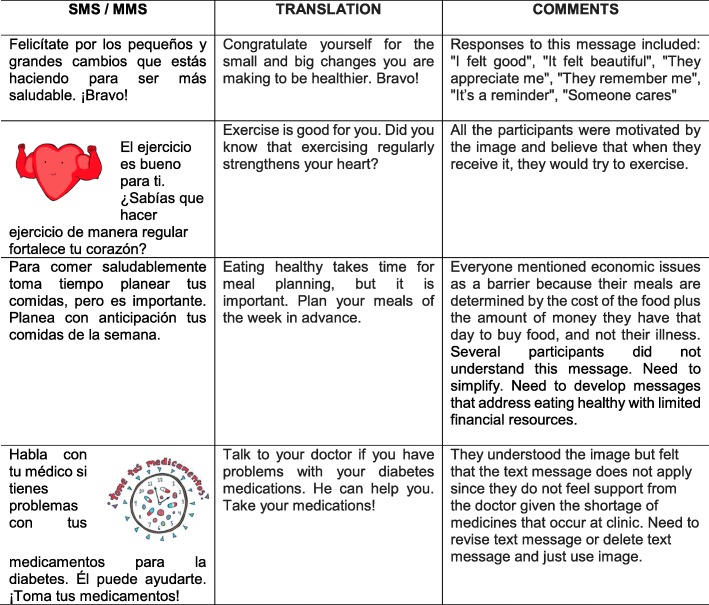
Examples of text messages and participant responses

We subsequently conducted a focus group with adults with T2D (at the same Seguro Popular clinics as the previous focus group) to determine their ability to receive and view text [short message service (SMS)] and picture messages [multimedia messaging service (MMS)] on different types of cell phones. Multimedia messaging (MMS) became standard in cell phone technology in 2011, which allows pictures to be received even if the phone does not have a camera. Moderators of the focus group sent text and image messages to all participants to determine their ability to receive the messages. We sent both low and high-resolution images to determine if that made a difference in receiving MMS messages. Moderators then recorded the participant’s ability to receive SMS and MMS messages.

There were seven adults with T2D who participated in this aspect of the text messaging evaluation with the following characteristics: 71.43% female, mean age 51.14 years (SD = 10.96), median age 50 years (IQR 20), and 100% had less than high school education and were married or in cohabitation, with a mean A1C 9.6% (SD = 1.13) and a median of three comorbidities. Participants had a wide variety of cell phone brands/models and used two different cell phone carriers. All participants were able to receive SMS messages without difficulty. However, many participants had difficulty receiving the MMS messages including not receiving the message, difficulty in downloading the message, or difficulty in viewing the image (too small). Participants who were able to receive messages preferred the high-resolution image as the colors were brighter and easier to view words and images. The results of this focus group reinforced the need to provide all participants with a color booklet with each text message to assure that the content of the text message was received by all participants. We also determined that we would send high-resolution image messages to enhance the visual experience as there was no difference in the ability to download images based on image size.

### Phase 5: Development of program manual

At the conclusion of the focus groups, we finalized the protocol for the *¡Sí, Yo Puedo Vivir Sano Con Diabetes!* program. The program consisted of seven group-based sessions, daily text messages, and monthly booklets that had each text/image message numbered in the order that they would be received if accessible by text message. For the group-based sessions, we created a training manual for the leaders of the program, a participant workbook, colored posters to be used in sessions, and materials needed for sessions (e.g., cards with images of different foods). We also developed a fidelity checklist for program leaders to complete after each session.

Our final text message bank consisted of 181 messages. There were approximately 20–30 messages for each process of behavior change (e.g., action planning, maintenance self-efficacy) and 30 messages for each content topic (e.g., eating healthy, physical activity). There were 96 messages that were illustrated. We also created the text message color booklets on 4 × 4 cards, with a single text message on each card. Cards were numbered to align with the text message of the day and collated with a binder ring, so that participants could easily flip from one message to the next. Daily text and image messages were provided via the iMessage app™ for IOS (Apple) because Web-based platforms did not provide services in Mexican territory.

### Phase 6: Train health care professionals in program implementation

We developed a training program for the facilitators of the program (group sessions coordinators) on the program protocol—a registered nurse and a social worker. This training entailed self-study, interactive sessions, and role-playing which required 4 days of training. There was one session of the training that was provided through distance learning by the original developer of the ¡*Sí, Yo Puedo Controlar mi Diabetes!* program. Plans for monitoring program fidelity were also reviewed.

The training program consisted of content on the program, the theoretical underpinnings of the program, the pathophysiology and treatment of type 2 diabetes, and the social determinants of health in Mexico City. Each session of the curriculum was reviewed in detail and strategies to enhance the success of group-based education were presented. Role playing was utilized to practice providing encouragement, empathy, active listening, and critical thinking. Case studies were used to allow class leaders to get feedback on addressing challenging situations (e.g., the participant did not accomplish goals, participant expresses common misconceptions about T2D). Time was allotted every day for questions and conducting one of the relaxation exercises used in the curriculum. During the last day of the training session, class leaders presented one session to the research team, and feedback was provided on aspects that went well and areas to improve.

Plans for monitoring program fidelity during implementation were also reviewed during the training program. Class leaders were instructed to follow the program manual, providing the same content for all sessions and all participants. The fidelity checklist was discussed and class leaders were asked to complete this after every session. In addition, class leaders received ongoing coaching by a master trainer with the objective to maintain high levels of implementation fidelity, to perform and document adaptations to the class plan, and to keep class leaders motivated. The master trainer also attended some of the sessions to conduct passive observation and to provide positive feedback to class leaders. Based on these aspects, an implementation analysis will be performed and documented in the future.

## Discussion and conclusion

We used a systematic approach, formative research, and collaboration with stakeholders to adapt evidence-based DSME programs to an international context and system of care. We also used an established behavior change theory (HAPA model) to inform the development of illustrated text messaging to supplement the DSME program, an innovative aspect of this program. As we developed our program protocol, we specifically sought to address perceived challenges to T2D self-management, provide content that met the need of adults with T2D and providers, and develop a text messaging component that was accessible and acceptable to adults with T2D in Seguro Popular clinics. In addition, we addressed system challenges of delivering diabetes care in low- and middle-income countries, such as lack of administrative support, clinic staff shortages, lack of established guidelines, and limited resources [35, 36]. We developed collaborations with the Ministry of Health in Mexico and administrators of Seguro Popular clinics, identified nurses as a health care professional to provide leadership in the implementation of the program, included a training program and detailed protocol to standardize guidelines and program delivery, made plans to use technology as an adjunct to clinical care, and identified the need to provide blood glucose-monitoring supplies to study participants. Collectively, this systematic program development process has the potential to enhance the feasibility, acceptability, efficacy, and scale-up of the program.

To evaluate the program, we will use a randomized, controlled pilot study design in which 40 adults with T2D will be randomized to the *¡Sí, Yo Puedo Vivir Sano con Diabetes!* + mHealth or wait-list control condition with the hypothesis that clinical (A1C, body mass index, blood pressure, T2D self-management (diet, exercise, medication, blood glucose monitoring) and self-efficacy outcomes will be greater in *¡Sí, Yo Puedo Vivir Sano Con Diabetes!* + mHealth participants compared to the wait-list control condition at 3 and 6 months of follow-up. We also hypothesize that the *¡Sí, Yo Puedo Vivir Sano Con Diabetes!* + mHealth program will be feasible and acceptable to adults with T2D and providers and that fidelity of the program will be maintained.

## Data Availability

Datasets used and analyzed during this study are available from the corresponding author on reasonable request.

## Abbreviations

A1C: Glycated hemoglobin
CALMS-D: Community Health Worker Assisting Latinos Manage Stress and Diabetes
DIALBEST: Diabetes Among Latinos Best Practices Trial
DSME: Diabetes self-management education
GAM: Mutual-Help Groups (acronym in Spanish)
HAPA: Health Action Process Model
IQR: Interquartile range
MMS: Multimedia messaging service
SD: Standard deviation
SMS: Short message service
T2D: Type 2 diabetes

## References

[1] World Health Organization. World health statistics 2011. Geneva: WHO Press; 2011.

[2] OECD. Reviews of Health Systems: Mexico 2016. Paris: OECD Publishing; 2016.

[3] Arredondo A, Reyes G (2013). Health disparities from economic burden of diabetes in middle-income countries: evidence from México. PLoS One.

[4] Barquera S, Campos-Nonato I, Carlos A-S, Lopez-Ridaura R, Arredondo A, Rivera-Dommarco J (2013). Diabetes in Mexico: cost and management of diabetes and its complications and challenges for health policy. Glob Health.

[5] Villalpando S, de la Cruz V, Rojas R (2010). Prevalence and distribution of type 2 diabetes mellitus in Mexican adult population: a probabilistic survey. Salud Publica Mex.

[6] López-Amador K, Ocampo-Barrio P (2007). Patient beliefs on their disease, eating habits, physical activity, and treatment in a group of Mexican subjects with diabetes. Vol 9. Archivos en Medicina Familiar.

[7] Weller SC, Baer RD, de Alba Garcia JG, Salcedo Rocha AL (2013). Are differences between patient and provider explanatory models of diabetes associated with patient self-management and glycemic control?. J Health Care Poor Underserved.

[8] Vilar-Compte M, Bernal-Stuart A, Orta-Alemán D, Vargas-Bustamante A (2014). Needs assessment analysis for a diabetes management intervention for low-income older adults in Mexico City.

[9] Compeán Ortiz LG, Del Ángel PB, Reséndiz González E, Piñones Martínez S, González Quirarte NH, Berry DC (2016). Self-care behaviors and glycemic control in low-income adults in México with type 2 diabetes mellitus may have implications for patients of Mexican heritage living in the United States. Clin Nurs Res.

[10] Fort MP, Alvarado-Molina N, Peña L, Mendoza Montano C, Murrillo S, Martínez H (2013). Barriers and facilitating factors for disease self-management: a qualitative analysis of perceptions of patients receiving care for type 2 diabetes and/or hypertension in San José, Costa Rica and Tuxtla Gutiérrez, Mexico. BMC Fam Pract.

[11] Instituto Nacional de Salud Pública (2016). Encuesta Nacional de Salud y Nutrición 2016 [Database].

[12] Comisión Nacional de Protección Social en Salud/Seguro Popular (2012). Catálogo Universal de Servicios de Salud (CAUSES) 2012. Mexico: Secretaría de Salud.

[13] Flores-Hernández S, Saturno-Hernández PJ, Reyes-Morales H, Barrientos-Gutiérrez T, Villalpando S, Hernández-Ávila M (2015). Quality of diabetes care: the challenges of an increasing epidemic in Mexico. Results from Two National Health Surveys (2006 and 2012). PLoS One.

[14] Secretaría de Salud (2014). Programa de Acción Específico: Prevención y Control de la Diabetes Mellitus 2013-2018. Mexico.

[15] World Bank (2012). Information and Communications for Development 2012: Maximizing Mobile.

[16] Stewart J (2009). Global mobile - strategies for growth.

[17] Comunicado de prensa 179/19. En México hay 74.3 millones de usuarios de internet y 18.3 millones de hogares con conexión a este servicio: ENDUTIH 2018 [press release]. Mexico City: INEGI; 2019.

[18] Instituto Nacional de Estadística y Geografía (INEGI) (2018). Encuesta Nacional sobre Disponibilidad y Uso de Tecnologías de la Información en los Hogares 2018 (ENDUTIH).

[19] Instituto Nacional de Estadística y Geografía (INEGI) (2018). Encuesta Nacional sobre Disponibilidad y Uso de Tecnologías de la Información en los Hogares 2018 [Database].

[20] Peña-Purcell NC, Boggess MM, Jimenez N (2011). An empowerment-based diabetes self-management education program for Hispanic/Latinos: a quasi-experimental pilot study. Diabetes Educ.

[21] Pérez-Escamilla R, Damio G, Chhabra J (2015). Impact of a community health workers-led structured program on blood glucose control among latinos with type 2 diabetes: the DIALBEST trial. Diabetes Care.

[22] Wagner JA, Bermudez-Millan A, Damio G (2016). A randomized, controlled trial of a stress management intervention for Latinos with type 2 diabetes delivered by community health workers: outcomes for psychological wellbeing, glycemic control, and cortisol. Diabetes Res Clin Pract.

[23] Vilar-Compte M, Burrola-Méndez S, Lozano-Marrufo A, Pardo-Carrillo M (2019). Community intervention for type 2 diabetes management among low-socioeconomic older adults in Mexico City.

[24] Cruz-Montes A (2017). Percepción sobre el automonitoreo de la glucosa en un grupo de adultos mayores participantes en una intervención comunitaria sobre diabetes.

[25] Whittemore R, Vilar-Compte M, De La Cerda S (2019). Challenges to diabetes self-management for adults with type 2 diabetes in low-resource settings in Mexico City: a qualitative descriptive study. Int J Equity Health.

[26] Bandura A. Social foundations of thought and action: a social cognitive theory. Saddle River: Prentice Hall; 1986.

[27] Montaño D, Kasprzyk D. Theory of reasoned action, theory of planned behavior, and the integrated behavioral model. In: Glanz K, Rimer BK, Viswanath K, editors. Health Behavior: Theory, Research and Practice. San Francisco: 5th ed: Jossey-Bass; 2015.

[28] Zimmerman MA (1995). Psychological empowerment: issues and illustrations. Am J Community Psychol.

[29] Schwarzer R (2008). Modeling health behavior change: how to predict and modify the adoption and maintenance of health behaviors. Appl Psychol.

[30] Martinez-Brockman JL, Harari N, Segura-Pérez S, Goeschel L, Bozzi V, Pérez-Escamilla R (2018). Impact of the Lactation Advice Through Texting Can Help (LATCH) trial on time to first contact and exclusive breastfeeding among WIC participants. J Nutr Educ Behav.

[31] Martinez-Brockman JL, Shebl FM, Harari N, Pérez-Escamilla R (2017). An assessment of the social cognitive predictors of exclusive breastfeeding behavior using the Health Action Process Approach. Soc Sci Med.

[32] Schwarzer R, Handwrterbuch E (2012). Health Action Process Approach (HAPA). Gesundheitspsychologie von A bis Z.

[33] Schwarzer R, Lippke S, Luszczynska A (2011). Mechanisms of health behavior change in persons with chronic illness or disability: the Health Action Process Approach (HAPA). Rehabil Psychol.

[34] Houts PS, Doak CC, Doak LG, Loscalzo MJ (2006). The role of pictures in improving health communication: a review of research on attention, comprehension, recall, and adherence. Patient Educ Couns.

[35] Esterson YB, Carey M, Piette JD, Thomas N, Hawkins M (2014). A systematic review of innovative diabetes care models in low-and middle-income countries (LMICs). J Health Care Poor Underserved.

[36] Lerin PS (2017). Recursos institucionales para diabéticos mayahablantes de Tizimín (Yucatán). Carencias y logros en los Grupos de Ayuda Mutua (GAM). Revista Pueblos y Fronteras Digital.

